# Bacterium detected by gram stain and drug sensitivity in Chinese children with acute sinusitis

**DOI:** 10.1186/s12887-023-04178-9

**Published:** 2023-07-11

**Authors:** Yan Li, Yinhui Zeng, Haiqing Xiao, Wenlong Liu

**Affiliations:** Department of Otolaryngology, Guangzhou Women and Children’s Medical Center, Guangzhou Medical University, No. 9, Jinsui Road, Guangzhou, 510623 China

**Keywords:** Bacteriology, Antibiotic sensitivity, ARS, Children

## Abstract

**Background:**

Acute rhinosinusitis (ARS) is one of the common diseases of upper respiratory tract infection in children. Bacterial infection is a significant aggravating factor in pediatric ARS. In this research, our goal was to detected the bacterial flora and antibiotic sensitivity of ARS in Chinese children.

**Methods:**

We recruited 133 children with ARS between January 2020 and January 2022 from our hospital. Sinus secretion were collected and cultured for Gram stain as well as antimicrobial susceptibility tests.

**Results:**

*Moraxella catarrhalis*, *Staphylococcus aureus*, *Haemophilus influenzae*, *Streptococcus pneumoniae* and *Pseudomonas aeruginosa* were detected in order in children with ARS, of which 25% were negative for bacterial culture and 10% were positive for two strains. Amoxicillin and clavulanate potassium were useful for *Haemophilus influenzae*, *Streptococcus pneumoniae* and *Moraxella catarrhalis*. Quinolones are useful for *Staphylococcus aureus*, *Haemophilus influenzae*, *Streptococcus pneumoniae* and *Pseudomonas aeruginosa*.

**Conclusions:**

This research updates the proportion of ARS bacterial infection in children in southern China and the antibiotic sensitivity.

## Introduction

Acute rhinosinusitis (ARS) is one of the most common infections of upper respiratory tract both in children and adults [1]. It is reported that 5-10% of children with upper respiratory tract infection are complicated with acute sinusitis and 6-13% of children were diagnosed with sinusitis at the age of 3 [2]. Although pediatric ARS is common in outpatient clinics in China, there is a lack of relevant data on its epidemiology and economic burden. Both the mucosa of nasal passages and paranasal sinuses are involved in most ARS cases [3–5]. Viruses and pathogenic bacteria are often found in nasal secretion of ARS patients [6–8]. Generally, most ARS were caused by virus and aggravated by bacterial coinfection or secondary infection [9, 10].

Sinus puncture culture was believed to be the most accurate method for diagnosing ARS previously. However, recent meta-analysis found that endoscopic guided middle meatus samples may be more sensitive and less invasive for ARS compared with sinus puncture [11, 12]. Therefore, the samples were collected from middle meatus with the help of endoscopy.

In pediatric ARS, it was discovered that respiratory bacteria were present in 65% of the sinus secretions, while bacterial and viral agents were present in 32% of the secretions [13]. Rhinovirus and influenza virus were the most prevalent viruses, while *Haemophilus influenzae* and *Streptococcus pneumoniae* ranked first in bacterial species [13]. However, the microbial composition which contributed to ARS was not static. For example, popularization of vaccines in children can lead to the changes of the frequency and bacteriology of ARS [14]. Generally, children with uncomplicated ARS need only symptomatic treatment. Antibiotics were used when complications or concomitant disease were found as described in several guidelines [15].

The bacteriology of ARS has, however, barely been investigated, especially in the population of Chinese children. In this research, we aimed was to detect the bacterial proportion of middle meatus secretions and antibiotic sensitivity of these bacterium in Chinese children with ARS. Our data can provide bacteriology basis for antibiotic treatment of ARS.

## Method

### Patients

We recruited 133 children with ARS between January 2020 and January 2022 from Guangzhou Women and Children’s Medical Center in Guangzhou, China. The demographic information of the enrolled children was collected at the first interview. The inclusion criteria were as follows: two or more symptoms attack out of blue, one of nasal obstruction or runny nose and the other of facial pain/compression or reduction or anosmia lasting less than 12 weeks as described by European Position Paper on Rhinosinusitis (EPOS2020) [15]. The exclusion criteria were as follows: antibiotics treatment within 1 weeks before enrollment, nasal allergy or asthma, chronic nasal symptoms or polyps, and prior nasal surgery. Written informed consent was obtained from the parents of all children and children over 7 years of age before enrollment. The Ethics Committee of Guangzhou Women and Children’s Medical Center approved the conduct of this study.

### Bacterial culture and antibiotic sensitivity test

Middle meatus secretions were removed by swab guided by endoscopy and placed in tubes containing transport medium for further detection. For bacterial cultures, all samples were stained with Gram stain and plated on blood agar and chocolate agar and incubated at 35 °C for 24 h in an atmosphere containing 5% CO_2_. Pathogenic bacteria were identified using standard methods [16]. Antimicrobial susceptibility tests were done by the BD Phoenix System as described previously [17].

### Statistical analyses

SPSS 17.0 software was used for statistical analysis. The numeric variables were presented as total numbers, percentages, and mean ± standard deviation (SD) values. The differences between different groups were compared using χ2 test and SNK test. When P < 0.05, the difference indicated statistical significance.

## Results

### Demographic characteristics of children

The basic information of study subjects is summarized in Table 1. During the study period, 856 children were diagnosed as ARS in our center. A total of 133 children were enrolled, but only 75 male and 42 females aged from 1 to 8 years old completed sampling. Disease duration ranged from 10 days to 12 weeks. The color of nasal mucus presented as yellow (47.8%), white (5.2%), and yellow green (47%).

**Table 1:** Demographic and clinical data of children with ARS

Characteristics	
Sex ratio (male:female)	75:42
Age (year)	4.16 ± 1.25
Disease duration (weeks)	4.11 ± 2.67
Color of nasal mucus	
Yellow	56 (47.8%)
White	6 (5.2%)
Yellow green	55 (47%)
Symptoms	
Nasal blockage	85(72.6%)
Nasal discharge	77(65.8%)
Facial pain/pressure	28(23.9%)
Reduction/loss of smell	35(29.9%)
Headache	21(17.9%)

The data was presented with mean ± standard error

ARS, acute rhinosinusitis

### The bacteria distribution tested by GS

**Fig. 1:**
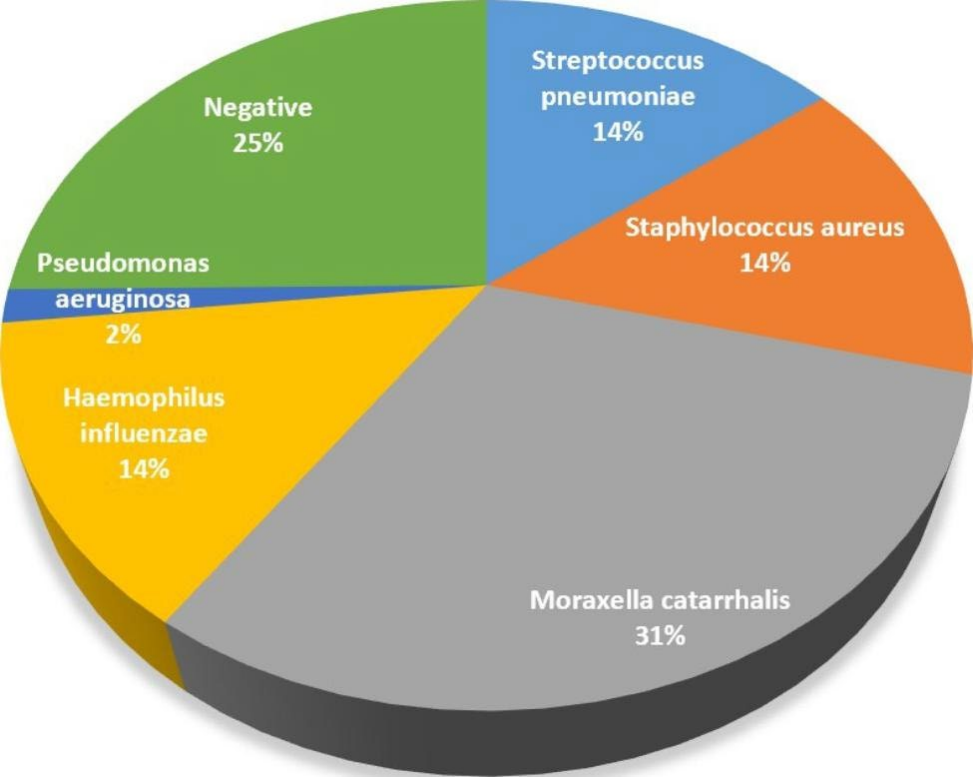
Proportions of bacteria detected in the sinus aspirates of pediatric patients with acute rhinosinusitis

The most common pathogens were Moraxella catarrhalis, *Staphylococcus aureus*, *Haemophilus influenzae*, *Streptococcus pneumoniae* and *Pseudomonas aeruginosa* in children with ARS. (Fig. 1). 33 cases showed as negative results in bacterial culture and 14 cases showed positive results of two strains.

### Antibiotic sensitivity test results

*Staphylococcus aureus* reacted well to quinolones, gentamicin and vancomycin. *Haemophilus influenzae* reacted well to amoxicillin and clavulanate potassium, second and third generation cephalosporin, macrolide, and quinolones. *Streptococcus pneumoniae* reacted well to amoxicillin and clavulanate potassium, third generation cephalosporin, macrolide, and quinolones. *Moraxella catarrhalis* reacted well to amoxicillin and clavulanate potassium and macrolide. *Pseudomonas aeruginosa* reacted well to third generation cephalosporin and quinolones (Table 2).

**Table 2:** Antibiotic sensitivity of common organism

Organism	Sensitivity rate							
	PN	AC	2nd	3rd	ML	QL	GT	VM
*Staphylococcus aureus* *Haemophilus influenzae* *Streptococcus pneumoniae*	4/18 - 4/21	- 17/18 17/21	- 18/21 -	- 21/21 18/21	7/18 21/21 0/21	18/18 21/21 21/21	16/18 - -	18/18 - 21/21
*Moraxella catarrhalis* *Pseudomonas aeruginosa*	- -	40/40 -	- -	- 2/2	35/40 -	- 2/2	- -	**-** **-**

All variables are expressed as number of sensitive organisms/number of resistant organisms

PN = penicillin; AC = amoxicillin and clavulanate potassium; 2nd = second generation cephalosporin; 3rd = third generation cephalosporin; ML = macrolide; QL = quinolones; GT = gentamicin; VM = vancomycin

## Discussion

ARS, one of the most common diseases in children, is increasingly prevalent. ARS causes annoying symptoms and affects quality of life negatively. Previous studies showed that 53.7% of ARS patients had bacterial infections [11]. *Streptococcus pneumoniae, Haemophilus influenzae*, and *Moraxella catarrhalis* are the most frequent bacterium in the world [18, 19]. However, the species of microbe vary depended on different circumstances such as age, season, and et al. [20, 21]. In children, the most frequent microbes in ABRS are *Streptococcus pneumoniae*, *Haemophilus influenzae*, *Moraxella catarrhalis*, *Streptococcus pyogenes*, and anaerobes [22].

For example, Brook et al. found that aerobes (mainly *staphylococci* and *streptococci*) were identified in about 38% of cases, whereas *Haemophilus* species were rare [23]. The most frequent bacteria were anaerobic Gram-positive cocci and Bacteroides species, especially *B. melaninogenicus*, and *fusobacteria*. Our data showed that 75% children with ARS can be diagnosed as acute bacterial rhinosinusitis (ABRS). *Moraxella catarrhalis, Staphylococcus aureus*, *Haemophilus influenzae*, *Streptococcus pneumoniae* and *Pseudomonas aeruginosa* were the most frequent microbes in our study. Moreover, about 10% cases is polymicrobial. Previous reports suggested that enteric bacteria and anaerobes are rarely identified except for odontogenic origin [24, 25]. Similarly, our study also did not identify enteric bacteria and anaerobes in children ABRS. The differences between our results and Brook’s study may be attributed to geographical and racial factors. Moreover, most studies conducted by Brook were at least ten years early and the microbiota may also change over time. Besides, the sample size may also affect our results.

Determining the antibiotic sensitivity helps to reduce the occurrence of side effects and chronicity as well as the development of resistance to the bacteria. Amoxicillin, amoxicillin-clavulanate or cephalosporins were recommended by the EPOS2020 for children with complications or concomitant disease that could be exacerbated by ARS [15]. Similarly, amoxicillin clavulanic acid, the second generation of cephalosporin and macrolide drugs are recommended by experts in China [26]. Our data showed that amoxicillin and clavulanate potassium, a first-line antibiotic recommend by several guidelines, are useful for *Moraxella catarrhalis, Streptococcus pneumoniae* as well as *Haemophilus influenzae.* While *Staphylococcus aureus, Haemophilus influenzae, Streptococcus pneumoniae* and *Pseudomonas aeruginosa* could be inhibited by quinolones. Indeed, previous studies identified quinolones as the drug of choice for the treatment of ARS in penicillin-allergic patients [27, 28], however, that quinolones often cause gastrointestinal symptoms, such as diarrhea and nausea. Further studies are needed to investigate the safety and efficacy of quinolones to determine whether they can be used as first-line drugs for the treatment of ARS.

Our study had some limitations. First, our study is a single center research, so our results cannot be generalized to other pediatric populations of China. Second, children with ARS should be tested for viruses to further expand the knowledge of ARS in follow-up studies. Further muti-center studies with large samples should be conducted to better understand the etiology of ARS in Chinese children.

Despite of the above limitations, our study reported the distribution of bacterial infections and the antibiotic sensitivity in pediatric ARS of our center, which is the largest children’s hospital with the most patients in South China. Our results provide a theoretical basis for a deeper understanding of the onset and clinical treatment of pediatric ARS.

## Data Availability

The datasets used and/or analysed during the current study are available from the corresponding author on reasonable request.
